# 

**Published:** 1897-02

**Authors:** 


ESTABLISHED 1855. SUBSCRIPTION, $2.00

### ATLANTA MEDICAL AND SURGICAL JOURNAL.

VOLUME XIII. NEW SERIES. ATLANTA, GA., FEBRUARY, 1897. NO. 12.

